# Differential connectivity between core hubs underlying demanding executive functions in schizophrenia compared to autism and control adults

**DOI:** 10.3389/fpsyt.2026.1757647

**Published:** 2026-02-20

**Authors:** Sofia Morais, Otília C. d’Almeida, Daniela Jardim Pereira, Alexandre Sayal, Bruno Direito, João Pereira, Salomé Caldeira, Sofia Meneses, Graça Areias, Vanessa Girão, Catarina Bettencourt, António Macedo, Miguel Castelo-Branco

**Affiliations:** 1 Psychiatry Department, Coimbra Local Health Unit, Coimbra, Portugal; 2 Faculty of Medicine, University of Coimbra, Coimbra, Portugal; 3 Coimbra Institute for Biomedical Imaging and Translational Research (CIBIT), University of Coimbra, Coimbra, Portugal; 4 Institute of Nuclear Sciences Applied to Health (ICNAS), University of Coimbra, Coimbra, Portugal; 5 Neurorradiology Functional Unit, Imaging Department, Coimbra Local Health Unit, Coimbra, Portugal; 6 Siemens Healthineers Portugal, Lisboa, Portugal; 7 Intelligent Systems Associate Laboratory (LASI), Guimarães, Portugal; 8 Center for Informatics and Systems of the University of Coimbra (CISUC), Coimbra, Portugal; 9 Faculty of Psychology and Neuroscience, Maastricht University, Maastricht, Netherlands; 10 Psychology Department, Coimbra Local Health Unit, Coimbra, Portugal; 11 Faculty of Psychological and Education Sciences, University of Coimbra, Coimbra, Portugal; 12 Centre for Research in Neuropsychology and Cognitive and Behavioral Intervention (CINEICC), Faculty of Psychological and Education Sciences, University of Coimbra, Coimbra, Portugal; 13 Institute of Psychological Medicine, Faculty of Medicine, University of Coimbra, Coimbra, Portugal

**Keywords:** autism spectrum disorder, executive function, functional connectivity, *n*-back, schizophrenia, working memory

## Abstract

**Introduction:**

Schizophrenia (SCZ) and autism spectrum disorder (ASD) are neurodevelopmental disorders with similar impairments in several neuropsychological domains, namely in executive function, hampering their differential diagnosis. We asked if brain activation and connectivity patterns within central nodes of the frontoparietal network (FPN), critical for executive control, are distinctively altered in these clinical populations during a working-memory task (*n*-back).

**Methods:**

Forty-five male adults (15 SCZ,15 ASD,15 controls) matched for age, education level, and handedness, underwent 3T brain fMRI during a *n*-back executive task. We functionally defined three core hubs of the FPN (primary outcome measure: dorsolateral prefrontal cortex -DLPFC and intraparietal sulcus -IPS), and the insula (secondary outcomes), a relevant connecting hub of the salience network (SN).

**Results:**

No significant differences were observed between SCZ and ASD. In contrast, we found significant connectivity differences which were higher for the SCZ group, particularly between the DLPFC-IPS and insula-IPS. Differences between SCZ and ASD dominated in the left hemisphere.

**Discussion:**

The distinct cortical activation and connectivity patterns in SCZ (increased connectivity within FPN and FPN-SN), as compared to ASD and controls, are consistent with a fundamental change in executive function in psychosis.

## Highlights

- SCZ individuals showed increased connectivity in the left hemisphere regarding DLPFC-IPS, compared to ASD.
- SCZ individuals showed increased connectivity in the right hemisphere regarding DLPFC-IPS, compared to controls.
- SCZ group showed increased connectivity in both hemispheres for the connection insula-IPS, compared to both groups.

## 1 Introduction

Executive functions comprise cognitive abilities that enable and drive adaptive goal-oriented behavior (1) through dynamic interactions among cortical networks (2). Working memory, one subdomain of executive function, is a limited capacity system that enables us to temporarily process, store, and manipulate information in conscious awareness (1). During tasks that involve working memory, the frontoparietal network (FPN) is usually activated (3). This network, also known as the central executive network, comprises two major hubs, the dorsolateral prefrontal cortex (DLPFC) and the posterior parietal cortex (4). FPN has a close relationship with the default mode (DMN) and the dorsal attention (DAN) networks (5). Within the FPN and shared with the DAN, the intraparietal sulcus (IPS) (6) is involved in working memory and numeric cognition (6). It is activated during response inhibition (7) and allows for FPN adjustment of adaptive control (2). The anterior insula has the capacity to gate executive control (8), and combined with the anterior cingulate cortex, plays a critical and causal role in the activation of FPN and deactivation of DMN (9). The salience network (SN), comprising the anterior insula and the dorsal anterior cingulate cortex, is involved in the dynamic switching between the FPN and DMN, allowing for disengaging from internal mental processes to respond to current goals (2). A strong negative correlation between activity patterns in the DMN and FPN has been associated with higher efficiency of executive function (10).

Schizophrenia (SCZ) and autism spectrum disorders (ASD) are two neurodevelopmental conditions often presenting overlapping symptoms. Executive function impairment was reported separately in SCZ (11) and ASD (12) but few studies compared executive function in both conditions. Using a comprehensive battery of neuropsychological tests, we previously found specific differences in the Digits Forward subtest scores between SCZ and ASD (13). We hypothesize that subtle changes in connectivity within the FPN may occur related to executive function impairment in these two prototypal disorders of executive dysfunction.

Some resting-state studies compared functional magnetic resonance imaging (fMRI) connectivity in both SCZ and ASD (14–20). However, these studies are not task based which limits the interpretation of cognitive alterations. Although valuable information can be obtained through resting-state studies, task-related studies are more suited to better differentiate executive function in these conditions, for example using n-back tasks. Few *n*-back functional connectivity studies were conducted in SCZ (21–23) and in ASD (24, 25). Only one study compared *n*-back functional connectivity in both conditions in adults, using magnetoencephalographic data (26). This study failed to detect differences in connectivity between the two groups, still, in ASD patients, working memory maintenance seemed to depend on the compensatory engagement of alpha-band oscillatory mechanisms in the FPN.

In a previous study from our group, after a real-time fMRI executive function training, using DLPFC as seed region in seed-based analysis, higher connectivity between DLPFC and other functionally related areas was found in ASD (27). These areas include the anterior insula, and IPS, related to the SN and FPN, respectively (28, 29). Therefore, our previous work (27) justifies the choice of our analysis on specific nodes of this network by comparing functional connectivity between the three groups: SCZ, ASD, and controls, using a demanding task (2-back) in terms executive load (30).

In the present study, we investigated the hypothesis that differential fMRI activation and connectivity can be found between adult males with SCZ, ASD, and controls matched for age, level of education, and handedness, targeting the frontoparietal network, as recruited by a working memory *n*-back task. The most relevant (primary) imaging metric was DLPFC–IPS connectivity and the secondary were Insula–DLPFC and Insula–IPS connectivity.

## 2 Materials and methods

### 2.1 Participants

We included outpatients with schizophrenia (SCZ, n=15) and autism spectrum disorder (ASD, n=15) from the Coimbra University Hospital, and a cohort of controls (n=15), matched for age, education level, and handedness. Clinical groups met the following inclusion criteria: (1) DSM-5 criteria for SCZ or ASD (31); (2) age between 18–40; (3) capacity to give consent; (4) and clinical stability in the last 6 months prior to enrollment. Participants with (1) medical or neurological comorbidity (e.g. epilepsy, head trauma, intellectual disability defined for Intelligence Quotient (IQ) <80); (2) substance abuse/dependence; (3) or contra-indications to magnetic resonance imaging, were excluded from the study.

Neuropsychological assessment was performed by a psychologist for the Wechsler Adult Intelligence Scale III (WAIS-III), Portuguese version (32) to evaluate intelligence (Full-scale-IQ, Verbal-IQ, and Performance-IQ); and the Autism Diagnostic Observation Schedule Second Edition (ADOS-2) Module 4 (33) to confirm the diagnosis of ASD group.

The Positive and Negative Syndrome Scale (PANSS) (34) was applied in SCZ group to measure the severity of their symptoms; and the Personal and Social Performance Scale (PSP) (35, 36) addressing psychosocial functioning, was performed by a psychiatrist.

In clinical groups, pharmacological exposure was calculated using the defined daily dose – DDD (37), and current antipsychotic exposure was calculated using chlorpromazine equivalents – CPZE (38, 39).

Control individuals were recruited from the community. A brief interview was conducted to exclude personal or family history of psychiatric disorders and general exclusion criteria. Handedness was determined through evaluation with the Edinburgh Handedness Inventory (40, 41). All subjects provided written informed consent for participation. This study was conducted in accordance with the Declaration of Helsinki and was approved by the local Ethics Committees of the Faculty of Medicine of the University of Coimbra (ref. CE-043/2020) and Coimbra Hospital and University Centre (ref. CHUC-109-18).

### 2.2 Resonance imaging data acquisition and paradigm description

All 45 participants were examined on a 3T Siemens Magnetom Prisma scanner (Siemens, Munich, Germany) with a phased array 64-channel birdcage head coil. The MRI session included a structural scan and a functional scan during an executive function task.

We acquired a 3D anatomical T1-weighted MPRAGE (magnetization-prepared rapid acquisition gradient echo) magnetic resonance imaging pulse sequence (TR = 2530 ms; TE = 3.42 ms; flip angle= 7°; 192 single-shot interleaved slices with no gap with isotropic voxel size 1 x 1 x 1 mm; FOV 256 mm) that lasted for 6 minutes. Functional imaging was acquired with an echo-planar imaging sequence (TR = 2000 ms; TE = 30 ms; flip angle= 75°; 32 slices in-plane resolution 3 x 3 mm; slice thickness 2.5 mm; slice gap 0.5 mm; FOV 192 x 210 mm).

**Figure 1:**
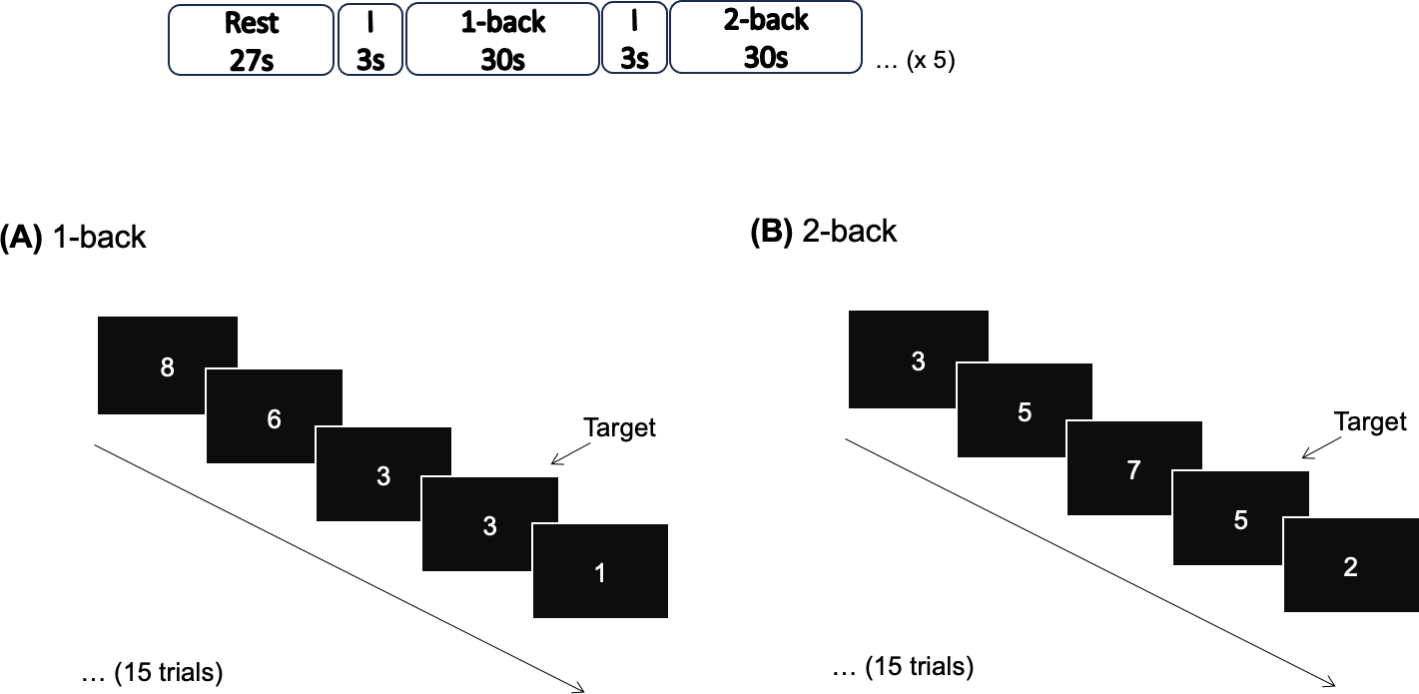
Diagram of the *n*-back task, displaying the two conditions **(A)** 1-back and **(B)** 2-back, alternately with Rest. I, Instruction; s, seconds.

*Executive function task.* To address working memory, an *n*-back task (**Figure 1**) was performed, including two conditions: 1-back and 2-back, interleaved with 27 seconds of rest (fixation cross) considered baseline, which was run by Presentation 20.1 (Neurobehavioral Systems, Inc.). Before each condition (1- or 2-back), the screen displayed the instruction (remember 1 or 2 numbers before), for 3 seconds. Participants viewed a sequence of 15 random numbers, each displayed for 400 milliseconds, and were instructed to press a button with their right index finger when the number displayed matched the one presented immediately before (1-back condition) or the two steps earlier in the sequence (2-back condition). Each sequence, which lasted 30 seconds, had 5 target numbers. Participants entered the scanner only after they understood the task and reached asymptotic performance levels (to prevent these effects from acting as a covariate on activation data). The *n*-back task comprised 5 trials, totaling 10.5 minutes in duration. Given that the subjects were pretrained before scanning they were at near ceiling level. The nature of these simple stimulus n-back tasks readily leads to ceiling performance (42, 43) precluding their use as analysis covariates.

### 2.3 Functional magnetic resonance imaging data preprocessing, contrast analysis, and region of interest selection

Imaging data was organized according to the Brain Imaging Data Structure (BIDS) using the Python package BIDSkit. Anatomical and functional data preprocessing was performed using fMRIPrep v23.0.2 (44) https://github.com/jmtyszka/bidskit. fMRIPrep’s preprocessing pipeline included: slice timing correction; motion correction; bias-field correction; normalization from subject space to the default Montreal Neurological Institute (MNI) space (MNI152NLin2009cAsym); and estimation of confound signals. Several confounding time-series were used based on the preprocessed BOLD including framewise displacement (FD). FD was computed using two formulations (absolute sum of relative motions, and relative root mean square displacement between affines). These were calculated for each functional run, both using the implementations in Nipype.

**Figure 2:**
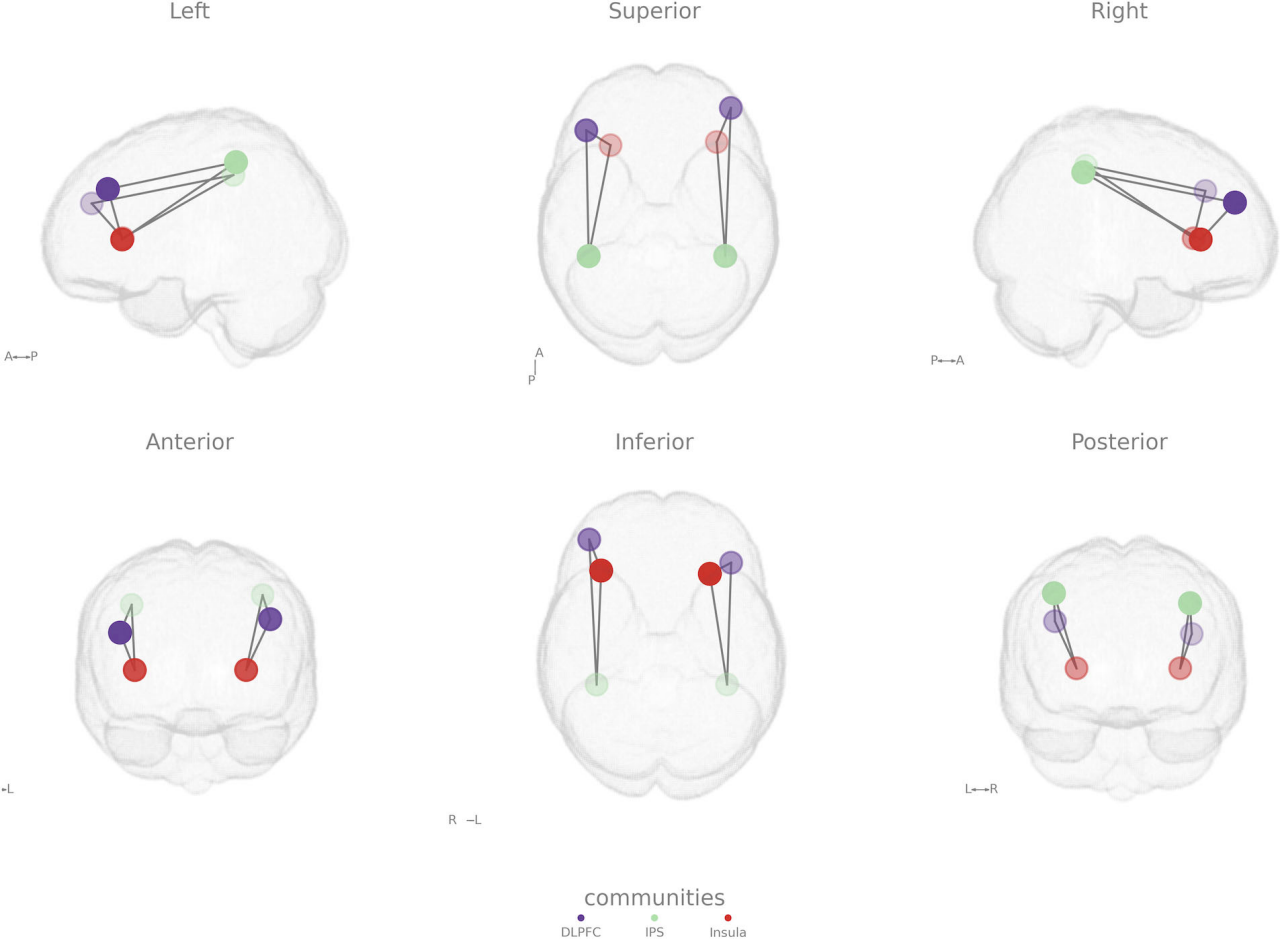
Representative scheme of the location of the core sites of frontoparietal– dorsolateral prefrontal cortex (purple sphere, DLPFC) and intraparietal sulcus (green sphere, IPS) – and salience – insula (red sphere) – cortical networks. The insula is strongly connected with other networks, namely the frontoparietal network (FPN) for regulating executive processes. R, right; L, left; A, anterior, P, posterior.

fMRI data were modeled with a block-related General Linear Model (GLM) analysis using the Python package Nilearn. The design matrix comprised separate predictors for each stimulus type (baseline; 1-back; 2-back), and 8 nuisance regressors (mean signal from the CSF, mean signal from white matter voxels, and the six head motion parameters). Second-level maps were obtained after estimating first-level models for all subjects and runs (n=45). The regions of interest (ROIs) were defined based on an effect across FPN for the ‘2-back task > baseline’ contrast, in the dorsolateral prefrontal (DLPFC) and insula. Given the challenge of isolating executive function in specific parietal regions we used the ‘2-back task > 1-back’ contrast, which allowed the delineation of a more circumscribed effort-related cluster, associated with higher cognitive load in the intraparietal sulcus (IPS). These ROIs were made spherical with an 8 mm radius and centered on the center of gravity of each cluster (**Figure 2**). The statistical threshold was set to p < 0.05 (Bonferroni-corrected) and the cluster threshold to k > 50. This definition was assisted by a neuroradiologist.

Mean beta values (mean changes in blood oxygen level dependent - BOLD activity), were extracted for each ROI, each hemisphere, and each aforementioned contrast. These values were then compared between each diagnostic group (SCZ, ASD, and controls).

### 2.4 Functional connectivity analysis

Functional connectivity analysis was performed in Nilearn. Time series for each of the 3 ROIs types (see **Figure 1**) were extracted using NiftiSpheresMasker with the following settings: high temporal filtering (0.008 Hz), detrending, confound regression (mean signal from the cerebrospinal fluid, mean signal from white-matter voxels, and the six head motion parameters), and normalization to z-scores. Each time course was then split into two conditions (1-back, 2-back) and baseline, according to the experimental protocol, considering an offset of 4 seconds to accommodate the hemodynamic delay.

Functional connectivity between ipsilateral ROIs of the major hubs of FPN and SN (DLPF-IPS, insula-DLPF, insula-IPS, **Figure 2**) was analyzed during the most demanding executive function task (2-back) comparing the z scores between groups, after Fisher’s z-transformation.

### 2.5 Statistical analysis

Statistical analyses were conducted in R Studio (v4.2.1). When data were normally distributed (Shapiro–Wilk test), parametric tests were used to test differences between groups. Otherwise, non-parametric Kruskal–Wallis H or Mann–Whitney U tests were applied. Fisher-Freeman-Halton’s exact test was used for categorical variables. Unless otherwise specified, *post-hoc* pairwise comparisons were corrected for multiple testing using Holm’s method.

For ROI analysis, beta values (‘2-back task > 1-back’) were averaged within each ROI for each hemisphere for each subject. Functional activation in the FPN hubs was evaluated with a two-way MANOVA (DLPFC and IPS) or two-way ANOVA (insula) with group (SCZ, ASD, control) and hemisphere as fixed factors. Significant multivariate effects were followed by univariate ANOVAs and *post-hoc* pairwise comparisons.

Connectivity between ipsilateral ROIs (DLPFC-IPS, insula-DLPFC, insula-IPS) was assessed using Fisher’s r-to-z transformed correlations during the 2-back task. Multivariate group differences were examined with MANOVA (Pillai’s trace), independently for each connection (both hemispheres), followed by univariate ANOVAs.

As a sensitivity analysis, group differences in mean beta values for the DLPFC and IPS, as well as in connectivity measures, were additionally evaluated using a permutation-based multivariate analysis of variance (PERMANOVA, with 5000 permutations based on Euclidean distances, vegan package). Homogeneity of multivariate dispersion was checked using permutation tests (coin package). Pairwise comparisons were performed as appropriate, and a fixed random seed (123) was used to ensure reproducibility.

For the SCZ group, the Spearman correlation between pharmacological exposure (calculated through defined daily dose – DDD) and particularly between antipsychotic exposure (calculated through chlorpromazine equivalents – CPZE), and neuroimaging results (beta values in contrast analysis, and z scores in functional connectivity) was calculated. We found no effects of medication on our results (further details are provided as Supplementary Material). Study design and data analysis were aligned with the Strengthening the Reporting of Observational Studies in Epidemiology (STROBE) consensus (45).

## 3 Results

### 3.1 Descriptive analysis

Demographic and clinical data are summarized in **Table 1**. As expected, the psychopharmacology and antipsychotic exposure was higher in patients with SCZ compared to ASD (p <0.001). All three groups did not significantly differ in age (p = 0.211), education (p = 0.914), laterality (p = 0.318), or psychosocial functioning (p = 0.683).

**Table 1:** Demographic and clinical data of study groups: schizophrenia (SCZ), autism spectrum disorder (ASD), and controls. Data is represented as mean ± standard deviation or absolute frequencies, as appropriate.

Characteristics	SCZ (n=15)	ASD (n=15)	Controls (n=15)	*p*-value
Sex (male/female)	15/0	15/0	15/0	–
Age (y)	26.3 ± 5.1	22.9 ± 4.9	25.8 ± 6.8	0.211
Education (y)	13.8 ± 2.6	13.6 ± 2.6	13.7 ± 2.2	0.914
Laterality (right/left)	13/2	15/0	15/0	0.318
Cigarette smoking (pack/year)	3.1 ± 3.9	0	4.4 ± 5.9	0.002
Pharmacological exposure, DDD (mg)	2.2 ± 1.5	0.3 ± 0.5	–	<0.001
Antipsychotic exposure, CPZE (mg)	353.2 ± 270.3	21.1 ± 42.9	–	<0.001
Duration of disease (y)	5.6 ± 4.1	–	–	–
FS-IQ	107.2 ± 11.9	109.4 ± 14.1	116.3 ± 12.4	0.085
Verbal-IQ	111.3 ± 13.4	111.8 ± 14.6	115.3 ± 14.7	0.704
Performance-IQ	101.1 ± 9.6	105.6 ± 15.4	114.1 ± 8.1	0.012
Functioning-PSP	69.5 ± 12.7	67.7 ± 7.0	–	0.683
PANSS	53.6 ± 11.6	–	–	–
ADOS-2	–	14.4 ± 4.8	–	–

DDD, defined daily dose; CPZE, chlorpromazine equivalents; FS-IQ, Full-Scale Intelligence Quotient; IQ, Intelligence Quotient; PSP, Personal and Social Performance Scale; PANSS, Positive and Negative Syndrome Scale; ADOS-2, Autism Diagnostic Observation Schedule Second Edition.

Possible ranges of scores: FS-IQ = [45, 155]; Verbal-IQ = [45, 155]; Performance-IQ = [46, 155]; PSP = [1, 100]; PANSS = [30, 210]; ADOS-2 = [0, 30].

In the SCZ group, patients were on stable antipsychotic medication, predominantly atypical antipsychotics: single second-generation (n = 7), single third-generation (n = 3), a combination of two second-generation (n = 2), or a combination of second and third-generation (n = 3) antipsychotic medication. Within the ASD group, 11 individuals were stable without any medication, 1 with a single second-generation antipsychotic medication, and 3 under a combination of antidepressant and second-generation antipsychotics. In the SCZ group, the mean disease duration was 5.6 ± 4.1 years.

Regarding Full-scale Intelligence Quotient evaluation, there were no statistically significant differences between the 3 groups (p = 0.085). However, there was a statistically significant difference in Performance-IQ, greater in controls compared to the SCZ group (p= 0.012).

Regarding clinical assessment, the SCZ group was assessed with PANSS (53.6 ± 11.6) and the ASD group with the ADOS-2 (14.4 ± 4.8).

Individuals in the ASD group had no exposure to cigarettes. No statistically significant differences were found between SCZ and the control groups in smoking.

### 3.2 Contrast analysis - strength of activation within the frontoparietal network in a numeric n-back contrast

**Figure 3:**
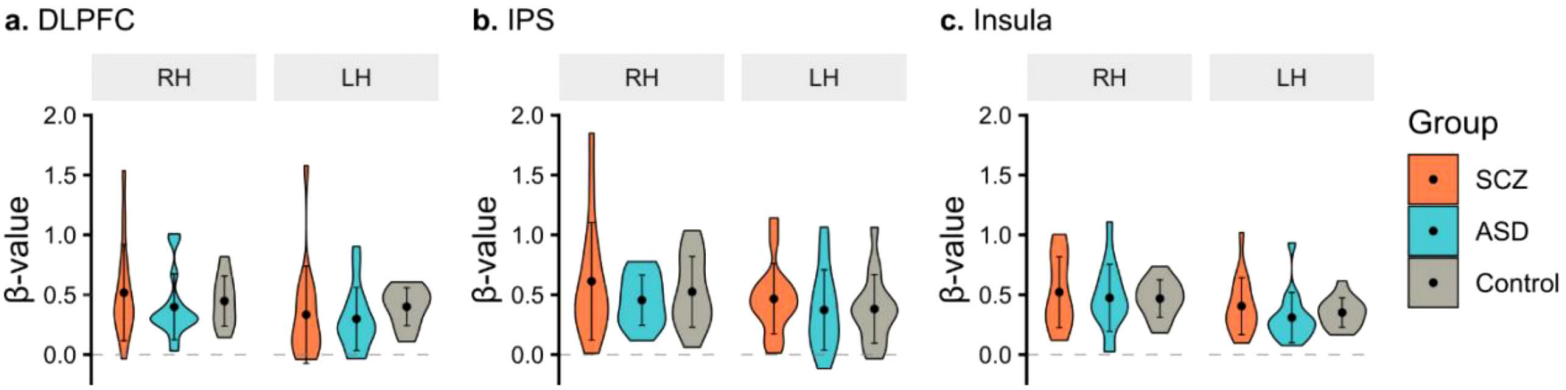
fMRI activation during the n-back contrast. For each group, schizophrenia (SCZ), autism spectrum disorder (ASD), and control, violin plots show the distribution of the average of β-value, reflecting the signal change, for each hemisphere of each subject within the regions of interest: **(A)** dorsolateral prefrontal cortex (DLPFC); **(B)** intraparietal sulcus (IPS); and **(C)** insula. The central dots represent the average value for each hemisphere of each group and error bars denote the standard deviation. RH, right hemisphere; LH, left hemisphere.

Beta values inside the ROIs of DLPFC, IPS, and insula in each hemisphere of each subject were averaged for the 2-back>baseline, 2-back>1-back, and 2-back>baseline contrasts, respectively (**Figure 3**). ROIs were centered on the coordinates MNI xyz (right DLPFC [44, 48, 26] and left DLPFC [-44, 34, 34], right IPS [40, -42, 44], and left IPS [-42, -42, 50], right insula [36, 28, 4], and left insula [-30, 26, 4]).

To evaluate between-groups differences in functional activation of the FPN hubs, a two-way MANOVA was conducted for DLPFC and IPS, with group and hemispheres as fixed factors. No statistically significant effects were found. Sensitivity analysis with non-parametric PERMANOVA showed similar results. Insula activation (2-back>baseline) was also compared between the three groups, as it plays a critical functional role between networks. No statistically significant interaction group*hemisphere, nor group difference were found. However, mean beta-values were significantly higher in the right insula (F(1,84) = 7.74, p = 0.007).

### 3.3 Functional connectivity analysis

#### 3.3.1 Frontoparietal network connectivity during a demanding executive function task (2-back)

**Figure 4:**
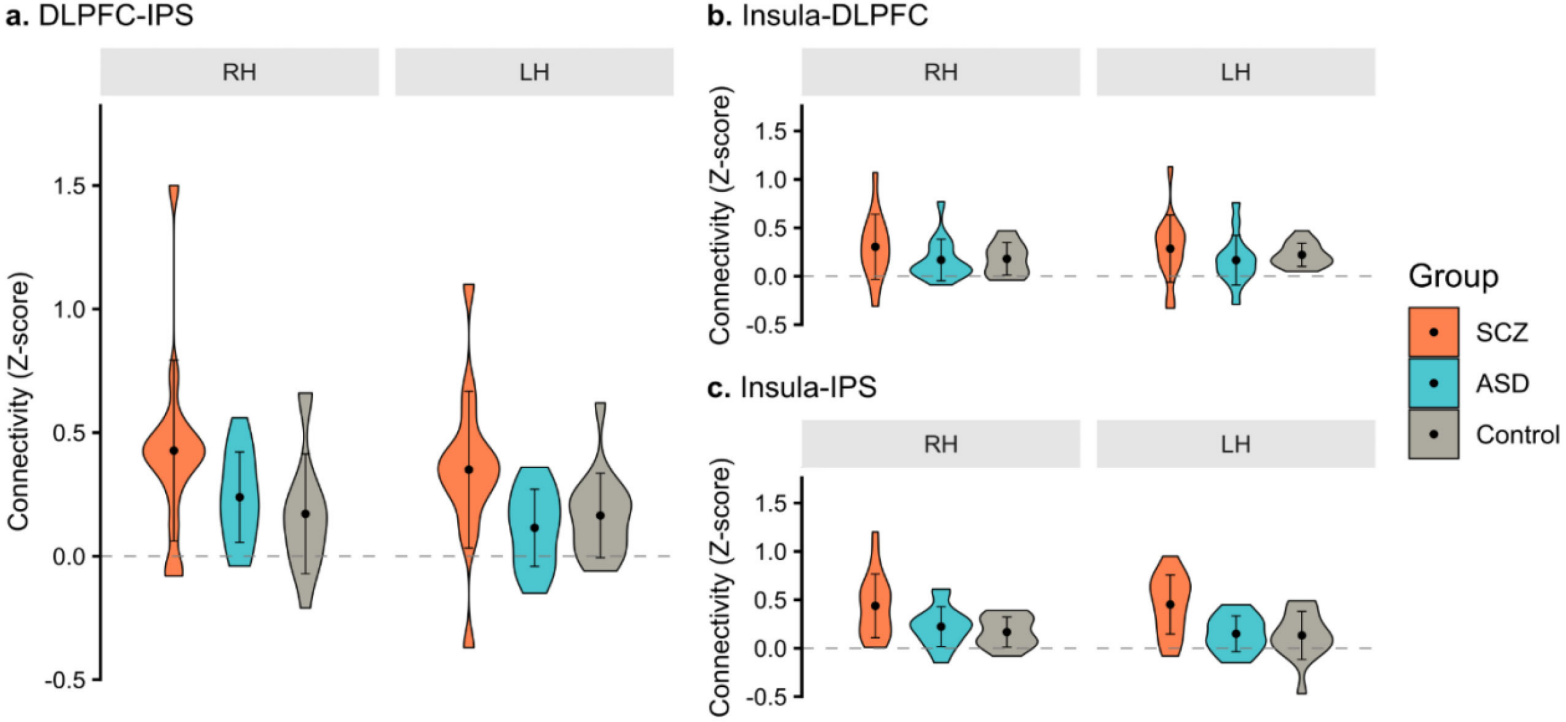
Functional connectivity (z scores) between key hubs of the **(A)** frontoparietal network – dorsolateral prefrontal cortex (DLPFC) and intraparietal sulcus (IPS), – and the **(B, C)** salience network (insula), during the 2-back task. For each group, schizophrenia (SCZ), autism spectrum disorder (ASD), and control, violin plots show the distribution of z scores, reflecting the connectivity strength, for each hemisphere, of each subject, between the ipsilateral regions of interest. Central dots represent the mean of z scores in each hemisphere of each group and the error bars denote the standard deviation. RH, right hemisphere; LH, left hemisphere.

To compare the connectivity between the major hubs of FPN between groups, a one-way MANOVA was conducted on z scores between DLPFC-IPS within each hemisphere during the most demanding task (2-back) (**Figure 4A**). We found a statistically significant multivariate effect of group in FPN connectivity (F(4, 84) = 2.82, p = 0.030, Pillai’s trace = 0.24). Follow-up univariate ANOVAs showed significant group differences in DLPFC-IPS connectivity in both hemispheres (right: F(2, 42) = 3.50, p = 0.039; left: F(2, 42) = 4.49, p = 0.017). Holm-corrected *post-hoc* comparisons showed that the differences in connectivity were statistically significant between the SCZ and control groups (p = 0.043) in the right hemisphere, and between SCZ and ASD groups (p = 0.021) in the left hemisphere.

Similar results were obtained with non-parametric PERMANOVA. A significant multivariate effect of group was found (F(2,42) = 3.90, p = 0.005). No significant differences in multivariate dispersion were observed (p = 0.395). Holm-corrected pairwise comparisons showed significant multivariate differences between the SCZ group and both the Control (p = 0.036) and ASD groups (p = 0.036).

#### 3.3.2 Salience network connectivity during a demanding executive function task (2-back)

Independent secondary analyses on the connection strength between the insula-DLPFC and the insula-IPS, for each hemisphere, between groups during the most demanding task (2-back), were also performed (**Figures 4B, C**). There were no statistically significant differences in the mean z score of the connection insula-DLPFC between groups (F(4, 84) = 0.79, p = 0.534, Pillai’s trace = 0.07). However, significant differences in the mean z score of the connection insula-IPS between groups were found (F(4, 84) = 3.57, p = 0.010, Pillai’s trace = 0.29). Follow-up analysis showed that these differences were present in both hemispheres (right: F(2, 42) = 5.26, p = 0.009; left: F(2, 42) = 7.68, p = 0.001). *Post-hoc* tests showed that, in the right hemisphere, the differences were statistically significant between SCZ and both ASD (p = 0.038) and control (p=0.011) groups. Similarly, in the left hemisphere, *post-hoc* tests showed significant differences between SCZ and both ASD (p = 0.004) and control (p=0.004).

Exploratory analysis with non-parametric PERMANOVA showed a similar pattern of results for each independent analysis. There was a significant multivariate effect of group (F(2,42) = 6.52, p = 0.001) when considering the insula-IPS connection of left and right hemispheres, but not for the insula-DLPFC connection analysis (F(2,42) = 1.07, p = 0.382). Considering the first, the group effects were driven by SCZ and both ASD (p = 0.010) and control (p = 0.009) differences. A statistically significant difference in multivariate dispersion was observed (p = 0.047), indicating that PERMANOVA results should be interpreted with caution.

## 4 Discussion

In this study, we investigated fMRI activity and connectivity between major nodes of the FPN and the SN while performing a demanding executive function task (*n*-back). Specifically, we asked if there are distinct patterns of activation and connectivity in these networks putatively recruited by a working memory task in adults with SCZ, ASD, and controls, matched for age, level of education, and handedness.

In the present study, we found higher activation for IPS in the SCZ group compared to controls, suggesting an increased recruitment of these regions to be able to carry out the task. The absence of activation differences regarding in ASD is consistent with prior work (25).

There is mounting interest in investigating the FPN connectivity, as neuroimaging-based brain patterns, in particular during the performance of an executive function task, may help to distinguish these conditions, in contrast with resting state studies. In particular, ASD without intellectual disability can be hard to differentiate from SCZ, given the large symptom overlap with SCZ, also often diagnosed in early adulthood.

Since SCZ and ASD seem to overlap in genetic (46), resting state and structural neuroimaging (19), clinical signs, and cognitive features (47), namely in relation to executive function, alternative approaches are required to provide a better distinction. Meta-analyses in SCZ (11) and in ASD (12) have shown impaired performance in executive function with neuropsychological evaluation in both conditions. This raises the question of whether there are distinct patterns of task-based connectivity between SCZ and ASD, and if the differences in connectivity underlie executive function deficits in adults with these conditions.

The major issue of most of the available fMRI studies is that they investigate connectivity without any task requirement which is in our view an inherent limitation of resting state studies (14–20).

According to our perspective, task-related studies provide more direct insights than resting-state approaches to directly investigate the functional significance of connectivity in executive circuits, in line with some authors that suggest the superiority of task-related measures such as predictive accuracy and reliability (48–50). Nevertheless, five meta-analyses of resting-state functional connectivity in SCZ (51–55) and one of task-functional connectivity in ASD (56) have shown abnormalities in both conditions. In SCZ, two meta-analyses described a disconnected large-scale brain networks model of SCZ (52, 53) in which the SN seems to play a core role (52). Other studies found that SCZ and the unaffected relatives have abnormal localized connectivity in the cerebellum (51) and suggested that resting-state functional connectivity holds a predictive biomarker of antipsychotic treatment response (54). Moreover, a recent meta-analysis revealed consistent and reliably aberrant functional connectivity in patients with SCZ (55). In ASD, one resting-state functional connectivity meta-analysis showed hypoconnectivity of the left amygdala, and the potential involvement of the fusiform gyrus and cerebellum in social cognition in ASD (56). It remains an open question on how these data compare and relate to task-based connectivity which may more directly relate to executive function.

In our study, in a demanding executive function task (2-back) we found that, in general, the SCZ group had higher connectivity in both DLPFC-IPS and insula-IPS. Interestingly, regarding hemisphere differences for SCZ vs. ASD vs. Controls, we found a laterality dissociation, namely a difference regarding FPN connectivity in the right hemisphere (SCZ vs Control) and a left SN-FPN difference SCZ vs. ASD. This right FPN difference may therefore be specific to general psychosis (SCZ vs Control), while the left SN-FPN alteration may be specific to the differentiation from ASD. Laterality plays as important role in working memory related DLPFC processing, with the left DLPFC sensitive to primarily verbal content, and right DLPFC sensitive to primarily non-verbal (visual and affective content) (57). The *n*-back task, being a numeric task, recruiting more largely the left hemisphere could explain our results.

Our pattern of results regarding ASD and SCZ is consistent with the prior work using working memory tasks, namely Garrity et al., 2007 (58) who reported increased functional connectivity involving components spanning temporal and frontal regions during an auditory oddball paradigm in schizophrenia. Meyer-Lindenberg et al., 2001 also investigated working memory connectivity (59). During a working memory (n-back) task, patients showed greater variability in expression of a task-related pattern during working memory, interpreted as difficulty sustaining a task-adequate network, which may explain why they need compensatory task related increases. Task related hypo-connectivity between frontal and parietal regions is consistent with the body of knowledge in autism spectrum disorder (ASD) (24). Just et al., 2007 reported that during an executive planning/problem-solving task (Tower of London), adults with ASD showed reduced functional connectivity between frontal and parietal regions, consistent with long-range “underconnectivity” (60). WM performance in ASD is therefore associated with reduced functional integration between frontal executive regions and posterior areas (often including parietal cortex).

Studies showed that the differences in executive function performance may rely on the dynamic interplay between the FPN and DAN, during cognitive and attention-demanding situations and these two with the DMN, activated during resting states. The switching between the FPN/DAN and the DMN is underlined by the SN. By hypothesis, changes in the connectivity between these networks may reflect working memory capacity or needs under cognitively demanding tasks.

Then, the increased connectivity found in SCZ in the right hemisphere for connection DLPFC-IPS compared to controls, and in the left hemisphere for the connection insula-IPS compared to ASD might reflect a connectivity-based compensatory mechanism although alternative explanations remain possible (27, 61).

Regarding the main limitation of the present study we have a relatively modest sample size, which is nevertheless the case in most task-based studies which are hard to perform in a multicentric fashion. Nevertheless, we were able to control for most relevant covariates. Medication effects cannot be fully excluded, especially in light of literature showing antipsychotic−related changes in connectivity and activation (62, 63). Dopamine blockade (especially D2/3 antagonism with many antipsychotics) can change WM-related frontoparietal function by altering the neural computations that make the network “efficient”. For example, Braun et al., 2021 (64) in a placebo-controlled crossover with amisulpride/risperidone (both D2-family antagonism profiles), reported changes consistent with higher “control energy” required to switch brain activation patterns during n-back after D2 blockade. This is essentially a network-level expression of reduced efficiency. Abott et al. noted that many acute D2-antagonism studies in healthy controls show reduced BOLD activation and that connectivity/coherence can change (63).

Also, we only studied male participants. Documented sex differences in working-memory (WM) activation and connectivity are usually small-to-moderate on average, highly overlapping between women and men, and being often task-, strategy-, and context-dependent (65, 66). In particular, Satterthwaite et al. (2014) reported that sex differences in functional network organization were observed and linked to cognitive measures, and that the connectivity architecture differed by sex in ways that map onto control networks often engaged by WM tasks (frontoparietal and related systems) (67).

In sum, we found a distinct cortical pattern of activation and connectivity in SCZ, as compared to ASD and controls. These results favor the view that ASD is mainly characterized by differences in cognitive style (68), while in SCZ, more fundamental changes in brain activity and connectivity are observed, consistently with the notion of a fundamental change in executive function in psychosis. A possible explanation could be the executive dysfunction more characteristic of SCZ, instead of the attentional deficit recognized in ASD, in which attentional difficulties are recognized as one of the most common comorbidities (69). Moreover, the co-occurrence of ASD and attentional problems is also associated with decreased adaptative skills (70).

## Data Availability

The raw data supporting the conclusions of this article will be made available by the authors, without undue reservation.
