# Supplementary material for: Differential connectivity between core hubs underlying demanding executive functions in schizophrenia compared to autism and control adults
Source: Front Psychiatry. 2026 Feb 20;17:1757647. doi: 10.3389/fpsyt.2026.1757647 (PMC12964060; doi:10.3389/fpsyt.2026.1757647)
Supplement: Supplementary file 1 [file DataSheet1.pdf]

Supplementary material

Medication dose effects on fMRI activation and connectivity in schizophrenia

For SCZ patients, there were no statistically significant correlations between pharmacological exposure (calculated through defined daily dose - DDD) or antipsychotic exposure (calculated through chlorpromazine equivalent - CPZE) dose and beta values in DLPFC, IPS, or insula in each hemisphere (Supplementary Figure 1, Supplementary Table 1).

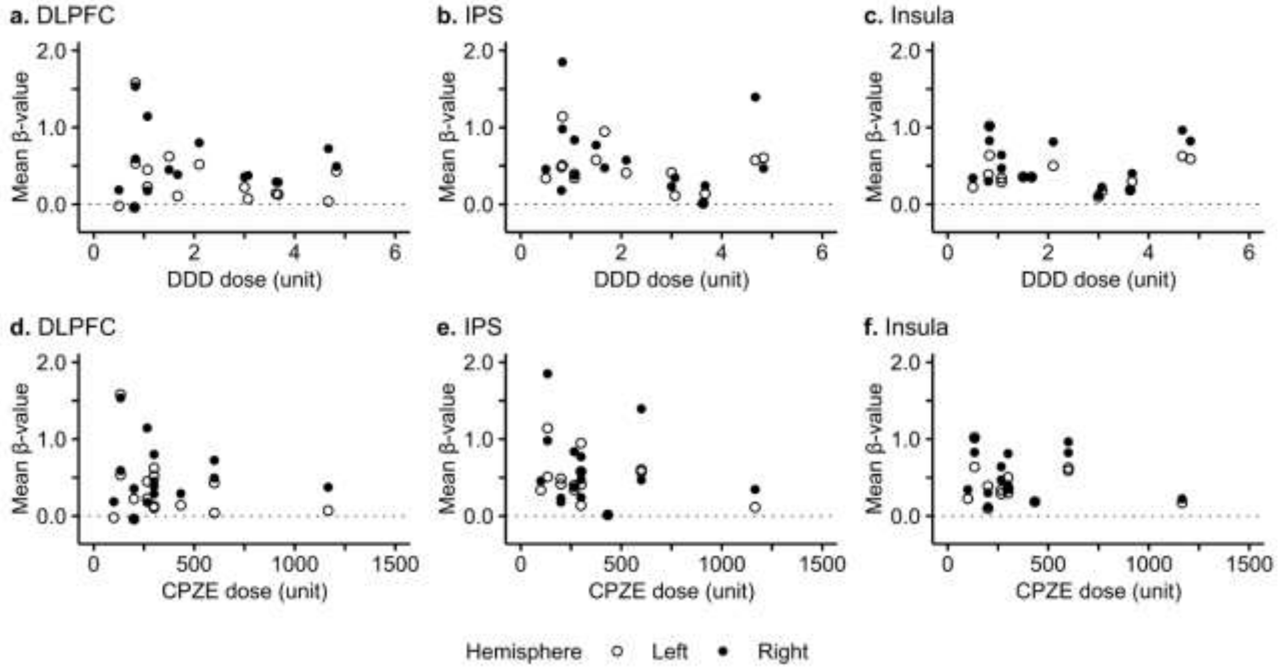

Supplementary Figure 1. Medication effects on fMRI activation. Scatterplots illustrate the relationship between the beta-value (β-value), reflecting the signal change within each region-of-interest and the pharmacological exposure – *(top row)* defined daily dose (DDD) and the antipsychotic exposure - *(bottom row)* chlorpromazine equivalent (CPZE) in schizophrenia group. Regions of interest include: **(A,D)** the dorsolateral prefrontal cortex (DLPFC), **(B,E)** the intraparietal sulcus (IPS), and **(C,F)** the insula. Closed and open circles represent the values for the right and the left hemisphere, respectively.

Also, no correlation was found between the z scores of the connections within DLPFC-IPS, Insula-DLPFC, or Insula-IPS, for either hemisphere and medication (DDD and CPZE dose) within the SCZ group (Supplementary Figure 2, Supplementary Table 1).

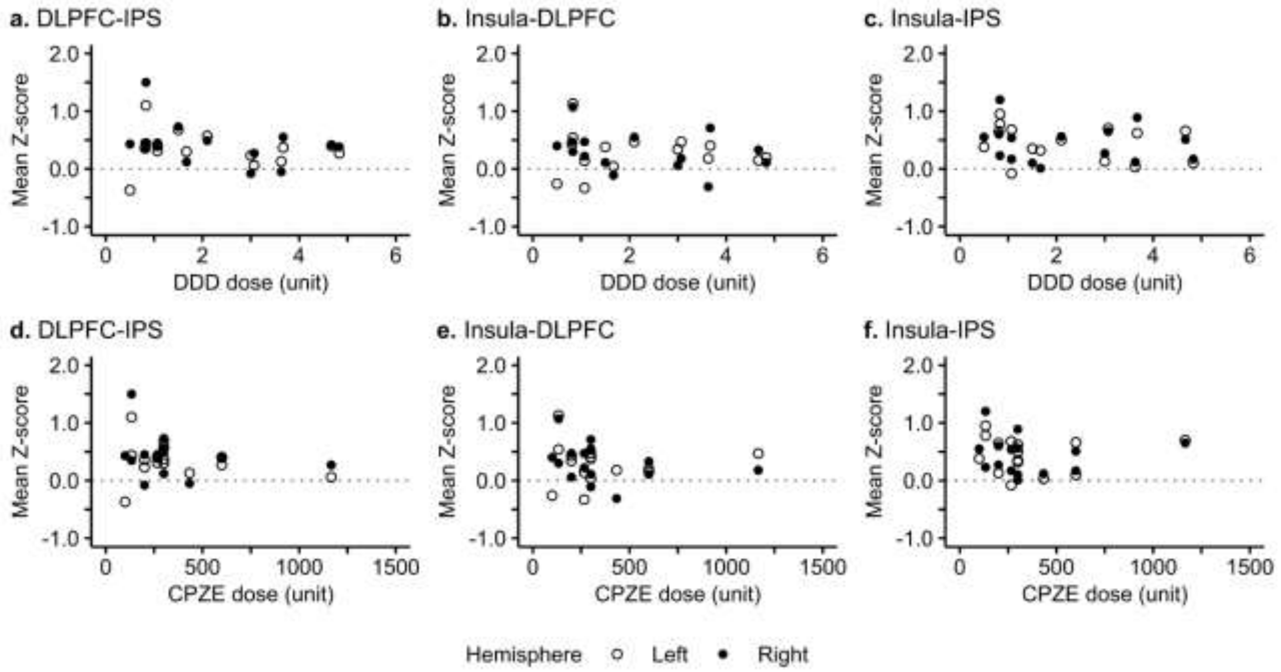

Supplementary Figure 2. Medication effects on functional connectivity. Scatterplots illustrating the relationship between the average z scores between the regions of interest (ROIs) and the pharmacological exposure – *(top row)* defined daily dose (DDD) and the pharmacological exposure - *(bottom row)* chlorpromazine equivalent (CPZE) in schizophrenia group. Connections include: **(A,D)** the dorsolateral prefrontal cortex (DLPFC) and the intraparietal sulcus (IPS), **(B,E)** the insula and DLPFC, and **(C,F)** the insula and IPS. Closed and open circles represent the values for the right and the left hemisphere, respectively.

Supplementary Table 1. Bivariate Spearman correlations between defined daily dose (DDD) and chlorpromazine equivalent (CPZE) and each target neuroimaging variable, namely mean β-values of the dorsolateral prefrontal cortex (DLPFC), intraparietal sulcus (IPS), and insula, as well as average z-scores of functional connectivity between DLPFC–IPS, insula–DLPFC and insula–IPS. Analyses were performed separately for the left and right hemispheres in patients with schizophrenia. 95% confidence intervals (95% CI) were estimated using bootstrapping resampling (n=2000) and p-values are uncorrected.

	**Left hemisphere**		**Right hemisphere**	
	**p-value**	**ρ (95% CI)**	**p-value**	**ρ (95% CI)**
**DDD vs β-values**				
**DLPFC**	0.66	0.13 [-0.53 ; 0.64]	0.76	-0.09 [-0.78 ; 0.5]
**IPS**	0.54	-0.17 [-0.78 ; 0.4]	0.71	-0.1 [-0.65 ; 0.5]
**Insula**	0.98	-0.01 [-0.6 ; 0.54]	0.66	-0.12 [-0.66 ; 0.5]
**CPZE vs β-values**				
**DLPFC**	0.71	0.1 [-0.49 ; 0.62]	0.55	-0.17 [-0.76 ; 0.44]
**IPS**	0.64	-0.13 [-0.65 ; 0.45]	0.63	-0.14 [-0.67 ; 0.51]
**Insula**	0.85	-0.05 [-0.61 ; 0.62]	0.71	-0.11 [-0.7 ; 0.58]
**DDD vs Z-scores**				
**DLPFC–IPS**	0.54	-0.17 [-0.71 ; 0.44]	0.26	-0.31 [-0.67 ; 0.17]
**Insula–DLPFC**	0.86	-0.05 [-0.62 ; 0.56]	0.18	-0.37 [-0.78 ; 0.16]
**Insula–IPS**	0.28	-0.3 [-0.74 ; 0.25]	0.55	-0.17 [-0.65 ; 0.38]
**CPZE vs Z-scores**				
**DLPFC–IPS**	0.55	-0.17 [-0.76 ; 0.52]	0.40	-0.23 [-0.64 ; 0.29]
**Insula–DLPFC**	0.79	-0.08 [-0.69 ; 0.53]	0.15	-0.39 [-0.71 ; 0.07]
**Insula–IPS**	0.45	-0.21 [-0.72 ; 0.43]	0.45	-0.21 [-0.66 ; 0.33]
